# Neurosteroid [3α,5α]-3-Hydroxy-pregnan-20-one Enhances the CX3CL1-CX3CR1 Pathway in the Brain of Alcohol-Preferring Rats with Sex-Specificity

**DOI:** 10.3390/life14070860

**Published:** 2024-07-09

**Authors:** Irina Balan, Adelina Grusca, Samantha Lucenell Chéry, Baylee R. Materia, Todd K. O’Buckley, A. Leslie Morrow

**Affiliations:** 1 Bowles Center for Alcohol Studies, School of Medicine, The University of North Carolina at Chapel Hill, Chapel Hill, NC 27599, USA; iirina@email.unc.edu (I.B.);; 2 Department of Psychiatry, School of Medicine, The University of North Carolina at Chapel Hill, Chapel Hill, NC 27599, USA; 3 Neuroscience Curriculum, The University of North Carolina at Chapel Hill, Chapel Hill, NC 27599, USA; 4 Department of Pharmacology, School of Medicine, The University of North Carolina at Chapel Hill, Chapel Hill, NC 27599, USA

**Keywords:** fractalkine, inflammation, allopregnanolone, chemokines, cytokines

## Abstract

This study investigates the impact of allopregnanolone ([3α,5α]3-hydroxypregnan-20-one or 3α,5α-tetrahydroprogesterone (3α,5α-THP); 10 mg/kg, IP) on fractalkine/CX3-C motif chemokine ligand 1 (CX3CL1) levels, associated signaling components, and markers for microglial and astrocytic cells in the nucleus accumbens (NAc) of male and female alcohol-preferring (P) rats. Previous research suggested that 3α,5α-THP enhances anti-inflammatory interleukin-10 (IL-10) cytokine production in the brains of male P rats, with no similar effect observed in females. This study reveals that 3α,5α-THP elevates CX3CL1 levels by 16% in the NAc of female P rats, with no significant changes observed in males. The increase in CX3CL1 levels induced by 3α,5α-THP was observed in females across multiple brain regions, including the NAc, amygdala, hypothalamus, and midbrain, while no significant effect was noted in males. Additionally, female P rats treated with 3α,5α-THP exhibited notable increases in CX3CL1 receptor (CX3CR1; 48%) and transforming growth factor-beta 1 (TGF-β1; 24%) levels, along with heightened activation (phosphorylation) of signal transducer and activator of transcription 1 (STAT1; 85%) in the NAc. Conversely, no similar alterations were observed in male P rats. Furthermore, 3α,5α-THP decreased glial fibrillary acidic protein (GFAP) levels by 19% in both female and male P rat NAc, without affecting microglial markers ionized calcium-binding adaptor molecule 1 (IBA1) and transmembrane protein 119 (TMEM119). These findings indicate that 3α,5α-THP enhances the CX3CL1/CX3CR1 pathway in the female P rat brain but not in males, primarily influencing astrocyte reactivity, with no observed effect on microglial activation.

## 1. Introduction

Neuroinflammation is increasingly recognized as a pivotal contributor to the onset and progression of neurological and psychiatric disorders [1,2,3,4]. This recognition has prompted research into potential therapeutic agents with the capacity to modulate neuroinflammatory processes. Among these agents, neuroactive steroids, such as allopregnanolone (also known as (3α,5α)3-hydroxypregnan-20-one or 3α,5α-tetrahydroprogesterone (3α,5α-THP)), have garnered significant attention. Studies have revealed a diverse array of action sites for these steroids, indicating that their multifaceted functions significantly contribute to their neurological effects and potential impact on central nervous system (CNS) disorders [5,6,7]. Moreover, 3α,5α-THP and other neurosteroids enhance inhibitory neurotransmission mediated by gamma-aminobutyric acid type A (GABA_A_) receptors, leading to anxiolysis, anti-convulsant activity and the enhancement of inhibitory circuits in the brain [8,9,10,11,12]. Importantly, the anti-inflammatory actions of neuroactive steroids are distinct from their GABAergic mechanisms [13,14,15]. The intravenous formulation of brexanolone (3α,5α-THP), a GABA_A_ receptor positive allosteric modulator, has been approved by the Food and Drug Administration (FDA) for the treatment of post-partum depression (PPD) [16]. Subsequent clinical observations have further indicated that brexanolone operates, at least in part, by suppressing the production of inflammatory mediators and alleviating the inflammatory responses triggered by toll-like receptor (TLR) signal activation in individuals suffering from PPD [17,18].

Research indicates that 3α,5α-THP, pregnenolone, and progesterone possess the ability to mitigate inflammatory responses triggered by TLR activation in various cell types and tissues, including the brain. This leads to a reduction in the production of inflammatory mediators, including tumor necrosis factor-alpha (TNF-α), interleukin-1 beta (IL-1β), IL-6, monocyte chemoattractant protein-1 (MCP-1), and high-mobility group box 1 protein (HMGB1) [13,14,17,19,20,21,22,23,24,25]. Moreover, additional research has shed light on the trophic and anti-inflammatory properties exhibited by 3α,5α-THP, revealing its ability to stimulate the production of brain-derived neurotrophic factor (BDNF) and IL-10 in male, but not female, rats [26,27,28].

Neuroinflammation is a complex process governed by intricate bidirectional neuron–glial signaling, in which fractalkine, alternatively referred to as CX3-C motif chemokine ligand 1 (CX3CL1), assumes a pivotal modulatory role. Produced by neurons, CX3CL1 undergoes a transition from a transmembrane protein to a soluble chemokine. It exerts its effects through its sole receptor, CX3CR1, predominantly expressed on microglial and astrocytic cells [29,30,31,32]. Within the CNS, CX3CL1 primarily functions to attenuate the pro-inflammatory response, with numerous studies demonstrating its neuroprotective and anti-inflammatory effects [33,34,35,36,37,38,39,40,41,42,43,44,45].

Interacting with CX3CR1 receptors on microglia, CX3CL1 plays a pivotal role in regulating various aspects of microglial physiology. A key function of CX3CL1 in neuron-microglia interactions is to suppress microglial activation [43,46,47,48,49]. For instance, in ethanol-induced brain injury, CX3CL1 guides microglia to clear apoptotic neurons by acting as a “find-me” signal, facilitating debris clearance and influencing inflammatory cytokine gene expression [50]. CX3CL1/CX3CR1 signaling diminishes the overproduction of inflammatory mediators, such as inducible nitric oxide synthase, IL-1β, TNF-α, and IL-6, thereby reducing microglial activation [35,36,38,47,48,49,51,52].

The disruption of CX3CL1/CX3CR1 signaling in the mouse brain results in a significant increase in glial fibrillary acidic protein (GFAP) expression [53]. Elevated levels of both GFAP mRNA and protein are characteristic of reactive astrocytes, a common feature observed across diverse CNS disorders and indicating an early response to injury. Notably, GFAP serves as a sensitive indicator, detectable even in scenarios where neuronal death is not apparent, suggesting synapse loss, minor demyelination, and the presence of extracellular amyloid-β oligomers [54,55,56]. The activation of GFAP causes a reduction in synaptic transmission and a simultaneous elevation in inflammatory and immune-related processes within the aging human brain [57]. Interestingly, GFAP shows promise as a reliable marker of subtle and diffuse structural brain damage in various neurological and systemic diseases, particularly in traumatic brain injury, that may not be easily detectable on imaging scans. Additionally, GFAP shows potential applications in predicting disability worsening in progressive multiple sclerosis, cognitive decline, and dementia progression in the elderly [58].

In selectively bred alcohol-preferring (P) rats, an innate reduction in brain CX3CL1 levels was observed, linked to the activation of inflammatory TLR4 signaling pathways [59]. Similarly, TLR4 inflammatory signal activation downregulates the expression of its receptor, CX3CR1, in microglia [47,60]. The reduction of CX3CL1 in P rat brains is associated with an imbalance favoring inflammatory modulators, such as MCP-1, over anti-inflammatory ones like CX3CL1 [59]. Previous research indicates that 3α,5α-THP mitigates MCP-1 levels by inhibiting inflammatory myeloid differentiation primary response 88-dependent TLR4 signaling and increases IL-10 production by enhancing anti-inflammatory toll/interleukin-1 receptor domain-containing adapter-inducing interferon-β (TRIF)-dependent TLR4 signaling in P rat brains [13,19,61]. Thus, 3α,5α-THP holds promise in preserving a balanced state between inflammatory and anti-inflammatory factors in the brain, potentially influencing susceptibility to inflammatory brain disorders. Furthermore, progesterone, a precursor of 3α,5α-THP, enhances CX3CL1/CX3CR1 signaling. This modulation effectively regulates harmful microglial activity, leading to a decrease in multiple pro-inflammatory cytokines and promoting increased neuroprotection within the retina [62].

While previous research has indicated the involvement of 3α,5α-THP in modulating inflammatory responses [13,14,19,20,61], its specific effect on CX3CL1 and associated signaling components in the brain remains unclear. Additionally, previous studies have demonstrated sex differences in the anti-inflammatory effects of neurosteroids [14,61]. A comprehensive understanding of these differences, especially concerning CX3CL1/CX3CR1 signaling within the brain, is crucial for tailoring therapeutic interventions precisely to address neurological disorders.

This study delves into the effects of 3α,5α-THP on CX3CL1, CX3CR1, phosphorylated (p) signal transducer and activator of transcription 1 (pSTAT1), transforming growth factor beta 1 (TGF-β1), and suppressor of cytokine signaling 3 (SOCS3) levels as well as the levels of ionized calcium-binding adapter molecule 1 (IBA1), transmembrane protein 119 (TMEM119), and GFAP as markers for microglia and astrocytes, respectively, in the brains of male and female P rats [53,54,63,64,65,66,67,68]. Additionally, we investigated the levels of cluster of differentiation (CD)68, CD36, triggering receptor expressed on myeloid cells 2 (TREM-2), and adipocyte complement-related protein of 30 kDa (Acrp30), all of which are pivotal in modulating brain inflammation. These proteins are primarily expressed in microglia within the brain [66,69,70,71].

3α,5α-THP is recognized for its regulatory role in cytokine and chemokine production, as well as their associated transcription factors [13,14,19]. Investigating the impact of 3α,5α-THP on STAT1 is crucial, given STAT1’s pivotal role in promoting CX3CL1 production. Studies have shown that overexpression of STAT1 enhances CX3CL1 promoter activity, thereby influencing CX3CL1 gene expression [68]. Additionally, understanding the influence of 3α,5α-THP on SOCS3 is warranted since the inhibition of SOCS3 function has been demonstrated to prevent CX3CL1 expression [72]. Notably, SOCS3/CX3CR1 double-knockout mice exhibited aggregated microglial activation and the degeneration of neuronal and epithelial cells in the retina [64]. Additionally, we investigated 3α,5α-THP effects on TGF-β1 since it has been shown to enhance CX3CR1 expression in rat microglial cells at both mRNA and protein levels, likely through augmented CX3CR1 gene transcription, leading to the suppression of glial cell activation [65].

This study demonstrates that the effect of 3α,5α-THP on CX3CL1 in P rat brains varies depending on both sex and brain region. Specifically, following 3α,5α-THP administration, females exhibit a significant increase in CX3CL1 levels in the nucleus accumbens (NAc), accompanied by elevated levels of CX3CR1, pSTAT1, and TGF-β1, indicative of enhanced anti-inflammatory signaling. In contrast, male P rats show negligible changes in CX3CL1, CX3CR1, pSTAT1, and TGF-β1 levels but exhibit elevation primarily in SOCS3 levels in the NAc after 3α,5α-THP treatment. Additionally, our findings extend beyond the NAc, demonstrating that in females, 3α,5α-THP increases CX3CL1 levels not only in the NAc but also in the amygdala, midbrain (including the periaqueductal gray and the raphe nuclei), and hypothalamus, with no significant alterations observed in males across these regions. Furthermore, there are no effects of 3α,5α-THP on CX3CL1 levels in the ventral tegmental area (VTA), hippocampus, and striatum in both males and females. Notably, both the male and female P rat NAc exhibit decreased GFAP levels without affecting microglial markers IBA1 and TMEM119 following 3α,5α-THP treatment. Moreover, 3α,5α-THP does not affect CD68, CD36, TREM-2, and Acrp30 levels in the male and female NAc, proteins that play important roles in modulating brain inflammation and are primarily found in microglial cells in the brain. This suggests an influence of 3α,5α-THP on astrocyte reactivity but not on microglial cell reactivity in the NAc. Overall, our study provides valuable insights into the therapeutic role of 3α,5α-THP in modulating neuroinflammation, with implications for the development of sex-specific and region-specific treatment strategies for neurological disorders. Further research is warranted to elucidate the underlying mechanisms and translate these findings into clinical applications.

## 2. Materials and Methods

### 2.1. Animals

Forty female and twenty male P rats, aged 3–4 months and weighing between 250 and 550 g, were used in these studies. The P rats were obtained from the Alcohol Research Center at Indiana University School of Medicine (Indianapolis, IN, USA) and subsequently bred in-house at the University of North Carolina School of Medicine (Chapel Hill, NC, USA). Rats were housed in pairs in Tecniplast cages (Tecniplast, West Chester, PA, USA) with corn cob bedding, maintained on a 12 h light–dark cycle (lights on at 0700 h), and provided with ad libitum access to food and water. Prior to experimentation, rats underwent a one-week period of acclimation to handling and injections to mitigate potential stress or anxiety, particularly during the administration of allopregnanolone or vehicle.

P rats, developed via selective breeding, serve as a model closely resembling individuals unexposed to alcohol but predisposed to seek and consume it. In contrast to their alcohol-non-preferring counterparts and wild-type rats, P rats exhibit voluntary alcohol intake and engage in binge drinking behavior [59,73,74,75,76]. The study aimed to investigate the regulation of CX3CL1/CX3CR1 signaling by 3α,5α-THP in the NAc of P rats. This interest stemmed from observations that selective breeding for alcohol preference resulted in the innate inhibition of CX3CL1 levels, and that 3α,5α-THP enhanced IL-10 levels in the NAc of P rats [59,61]. Moreover, the NAc is a critical brain region involved in the development, maintenance, and relapse of alcohol use disorders (AUDs), making it an important target for research and potential therapeutic interventions aimed at treating AUDs [77,78]. Additionally, CX3CL1 levels after treatments with 3α,5α-THP vs. vehicle control were examined in the amygdala, hypothalamus, VTA, hippocampus, and striatum, as well as the midbrain containing both the periaqueductal gray and the raphe nuclei. These brain regions, along with the NAc, collectively contribute to the complex neurobiology of AUDs, involving processes such as reward processing, stress regulation, memory formation, and habit formation. Dysfunction in any of these areas can influence susceptibility to AUDs and the progression of the disorder [59,75,76,77,78,79,80,81,82,83,84,85,86].

All experimental procedures adhered to NIH Guidelines and were approved by the Institutional Animal Care and Use Committee at the University of North Carolina (Chapel Hill, NC, USA). To mitigate potential circadian variations in neurosteroid levels, experiments were conducted in the morning [87,88]. The timing of experiments differed by 49 days between males and females.

For immunoblotting and enzyme-linked immunosorbent assay (ELISA) experiments, P rats were randomly divided into two groups: one receiving 3α,5α-THP (10 mg/kg) (males: *N* = 10; females: *N* = 10) and the other receiving a vehicle (45% *w*/*v* 2-hydroxypropyl-β-cyclodextrin) (males: *N* = 10; females: *N* = 10) via intraperitoneal (IP) injection. After 60 minutes, the rats were euthanized via decapitation. Their brains were harvested and stored at −80 °C. Using a rat brain matrix, six 2 mm thick coronal brain sections were prepared. The initial incision was made at the optic chiasm, followed by two rostral sections and four caudal sections from this starting point. The NAc, amygdala, hypothalamus, VTA, hippocampus, and striatum, as well as the midbrain containing both the periaqueductal gray and the raphe nuclei, were precisely dissected from these slices utilizing established neuroanatomical landmarks [89].

Twenty female P rats were randomly selected for immunofluorescence tissue staining, with half of the rats (N = 10) receiving 3α,5α-THP treatment and the other half (N = 10) receiving vehicle control treatment. The choice of dosage and timing was informed by previous studies indicating anti-inflammatory, anticonvulsant, and anxiolytic properties, without inducing hypnotic effects [90,91,92].

### 2.2. Tissue Lysate Preparation for Immunoblotting and ELISA

For immunoblotting and ELISA analysis of whole tissue lysates, brain tissues were dissected and lysed using CelLytic MT solution (Sigma Aldrich, St. Louis, MO, USA, Cat. # C3228), supplemented with protease and phosphatase inhibitor cocktails from Sigma Aldrich (St. Louis, MO, USA).

Following lysis, the samples were sonicated twice for 30 s using a Sonicator ultrasonic processor (Misonix, Inc., Farmingdale, NY, USA), followed by centrifugation at 15,000× *g* at 4 °C for 30 min. Total protein levels were quantified using the bicinchoninic acid assay (BCA) kit from Thermo Fisher Scientific (Waltham, MA, USA, Cat.# 23228 and Cat.# 1859078).

### 2.3. Immunoblotting

Immunoblotting assays were performed as previously described [61]. The protein samples (35 μg/lane) were denatured at 95 °C for 5 min using lithium dodecyl sulfate (LDS) sample buffer (Cat.# NP0007, Thermo Fisher Scientific) supplemented with sample reducing agent (Cat.# NP0009, Thermo Fisher Scientific, Waltham, MA, USA). Electrophoresis was performed on NuPAGE™ 10% Bis-Tris Midi Protein Gels (Cat.## WG1202 and WG1203, Thermo Fisher Scientific, Waltham, MA, USA) at an initial voltage of 125 V for 10 min, followed by 165 V for the remaining duration. After electrophoresis, the separated proteins were transferred onto a polyvinylidene difluoride membrane (PVDF; Cat.# IB24001, Thermo Fisher Scientific, Waltham, MA, USA).

The membranes were blocked for 2 h at room temperature using either a 5% solution of blotting-grade blocker (Cat.# 1706404, Bio-Rad, Hercules, CA, USA) or 5% bovine serum albumin (BSA) for phosphorylated primary antibodies. Subsequently, the membranes were incubated overnight at 4 °C with primary antibodies, followed by a 1 h incubation at room temperature with horseradish peroxidase-conjugated secondary antibodies. Both primary and secondary antibodies were appropriately diluted in either a 5% blotting-grade blocker buffer or 5% BSA for phosphorylated primary antibodies.

After the antibody incubation steps, the membranes were washed three times for 10 min each in Tris-buffered saline supplemented with 0.05% Tween-20 (TNT). Immunoreactive bands were visualized using the PlusECL kit reagents (Cat.# NEL105001EA, Perkin Elmer, Waltham, MA, USA), and chemiluminescent signal detection was performed using the ImageQuant LAS4000 system (Cytiva, Marlborough, MA, USA). Membrane images were analyzed using ImageQuant TL version 8.1.0.0 software (Cytiva, Marlborough, MA, USA).

Densitometric measurements were normalized by dividing each value by the corresponding β-actin densitometric measurement. The obtained values are presented as percentages relative to the average value of the vehicle control, along with the corresponding standard error of the mean (SEM).

### 2.4. ELISA

Enzyme-linked immunosorbent assays (ELISA) were performed on protein extracts using ELISA kit (Raybiotech, Norcross, GA, USA) designed for CX3CL1 (Cat. # Q6IRF7), following the manufacturer’s guidelines. The outcomes are presented in picograms per milligram of total protein (pg/mg).

### 2.5. Tissue Section Preparation, Immunofluorescence Staining, and Microscopy

P rats were anesthetized using the isoflurane drop method and transcardially perfused with 1× phosphate-buffered saline (PBS) followed by 4% paraformaldehyde. Brains were then stored in 4% paraformaldehyde for 24 h at 4 °C and transferred to 30% sucrose at 4 °C until fully submerged. Coronal brain sections (40 μm) were obtained using a freezing microtome and stored in cryoprotectant at −30 °C until immunofluorescent staining.

For the double-immunofluorescent staining of CX3CL1 alongside neuronal nuclear protein (NeuN), TMEM119, and GFAP, three coronal sections within the NAc, each 40 μm thick and selected from a 1:6 series with an approximate spacing of 240 μm between each, were used. Sections underwent washing in 1× PBS, repeated three times, and were then treated with Antigen Retrieval Citra (Biogenex Laboratories, Fremont, CA, USA) for 1 h at 70 °C. Subsequently, sections were blocked in 10% normal goat serum (Vector Laboratories, Newark, CA, USA) for 1 h at room temperature and incubated with primary antibodies (Table 1) at 4 °C for 72 h. After washing, sections were incubated with secondary antibodies (Alexa Fluor 488 or 594 dye) (Thermo Fisher Scientific, Waltham, MA, USA) for 2 h at room temperature away from light. Following another round of washing, sections were mounted and cover-slipped with Prolong Gold Antifade Mounting media (Thermo Fisher Scientific, Waltham, MA, USA) and dried for 24 h. Slides were then stored at 4 °C until imaging. Imaging was performed on the Olympus FV3000RS microscope (Olympus Corporation of the Americas, Breinigsville, PA, USA) at a magnification of 20× and double zoom (40×) with an average of 8–5 frames acquired at a sampling speed of 2 µs/pixel. Z-stacks were acquired with a z step of 0.91 µm/slice. Laser signals for Alexa Fluor 488 and Alexa Fluor 594 were utilized, with power maintained below 10%. Images obtained were later extracted as .tiff files and imported into NIS Elements (Nikon Instruments Inc., Melville, NY, USA) for qualitative analysis. Cellular localization of CX3CL1 within neurons (NeuN), microglia (TMEM119), and astrocytes (GFAP) was examined.

### 2.6. Antibodies

Antibodies were procured commercially and utilized according to the manufacturer’s instructions. Details of primary antibodies, host species, clonality, and dilutions are provided in Table 1. The horseradish peroxidase-labeled secondary antibodies were anti-rabbit (Cat. # 7074, Cell Signaling Technology, Danvers, MA, USA) and anti-mouse (Cat# 7076, Cell Signaling Technology, Danvers, MA, USA).

### 2.7. Statistics

Due to differences in experiment timing between males and females, separate studies were conducted for each sex. In each experimental group of P rats, protein levels between neurosteroid and vehicle control treatments were compared. Each group included two datasets (vehicle vs. neurosteroid), allowing for either a parametric unpaired *t*-test or a nonparametric Mann–Whitney U test. The data distribution was assessed using the Shapiro–Wilk normality test. For normally distributed data, an unpaired *t*-test was employed, examining the resulting t-value, degrees of freedom (df), and significance level (*p*-value). The t-value reflects the difference between group means relative to within-group variability. The degrees of freedom (df) are calculated based on the sample sizes of the two groups being compared. In cases of non-normally distributed data, the Mann–Whitney U test was utilized, analyzing the resulting U-value, sample size (n), and *p*-value. The U-value represents the combined rank order of data points in the two independent groups, indicating significant differences in their distributions. A lower U-value suggests one group tends to have lower values, while a higher U-value indicates the opposite. The U-value’s significance is determined by calculating a *p*-value. Statistical analysis was performed using GraphPad Prism 9.5.1 (733) software (GraphPad Software, LLC, Boston, MA, USA), with a significance level set at *p* < 0.05. The data are presented as mean values ± SEM, expressed as a percentage for immunoblotting assays or in pg/mg for ELISA assays.

## 3. Results

### 3.1. 3α,5α-THP Upregulates the Levels of CX3CL1 as Well as CX3CR1, pSTAT1, and TGF-β1 in the NAc of Female, but Not Male, P Rats

**Figure 1:**
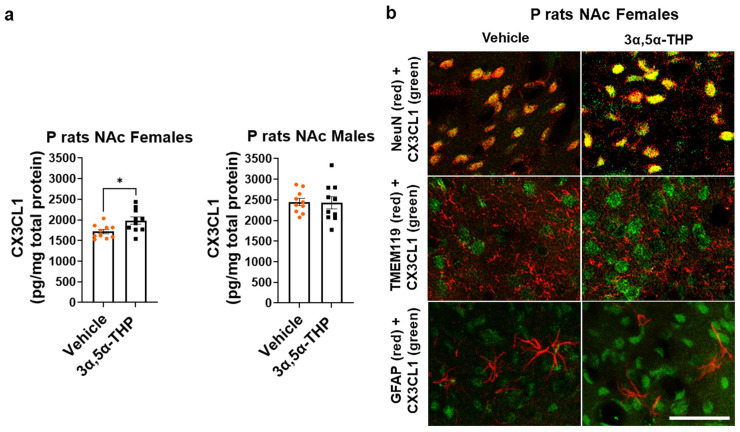
(**a**) 3α,5α-THP upregulates the levels of CX3-C motif chemokine ligand 1/fractalkine (CX3CL1) in the nucleus accumbens (NAc) of female, but not male alcohol-preferring (P) rats. Male and female P rats (n = 10/group) were treated intraperitoneally with 3α,5α-THP (10 mg/kg) or vehicle (45% *w*/*v* 2-hydroxypropyl-β-cyclodextrin) control. After 60 min, the nucleus accumbens (NAc) was examined using ELISA to determine CX3CL1 expression. In females, the administration of 3α,5α-THP resulted in a significant increase in CX3CL1 levels within the NAc (*p* = 0.02). However, in male rats, 3α,5α-THP treatment did not lead to a notable change in CX3CL1 expression within the NAc (*p* = 0.91). In the graphs, every column, along with its error bar, represents the mean ± SEM, expressed in pg/mg of total protein level. Each circle represents an individual CX3CL1 value for vehicle-treated rats, while the black squares indicate the CX3CL1 values for the 3α,5α-THP-treated rats. * *p* < 0.05. (**b**) Qualitative evaluation of 3α,5α-THP’s impact on the intracellular distribution of CX3CL1 in the NAc of female P rats. Double-immunofluorescent staining was conducted using antibodies targeting CX3CL1 alongside NeuN (a neuronal marker), TMEM119 (a microglial marker) or GFAP (an astrocyte marker). In vehicle control, CX3CL1 was observed to localize within NeuN-positive neuronal cells while not co-localizing with TMEM119-positive microglial cells or GFAP-positive astrocytic cells. Treatment with 3α,5α-THP did not induce any evident alterations in CX3CL1’s intracellular localization. Scale bar is 50 µm.

In our previous studies, we demonstrated that 3α,5α-THP enhances anti-inflammatory IL-10 cytokine production via the endosomal TRIF-dependent TLR4 signaling pathway in the brain of P rats. However, this upregulation occurs in males, but not females [61]. The baseline levels of the anti-inflammatory chemokine CX3CL1 are innately downregulated in the brains of P rats [59]. In this study, we further examined the impact of 3α,5α-THP (10 mg/kg, IP) on the levels of CX3CL1 in the NAc as well as the amygdala, midbrain (containing both the periaqueductal gray and the raphe nuclei), hypothalamus, VTA, hippocampus, and striatum in both male and female P rats. In females, the administration of 3α,5α-THP led to a statistically significant increase in CX3CL1 levels in the NAc (+15.6 ± 5.9%; *t*-test: t = 2.61, df = 18, n = 10, *p* = 0.02) (Figure 1a). In contrast, 3α,5α-THP treatment of males did not exert a significant effect on CX3CL1 expression in the NAc (*t*-test: t = 0.12, df = 18, n = 10, *p* = 0.91) (Figure 1a). Moreover, 3α,5α-THP administration upregulated CX3CL1 levels in the amygdala, midbrain, and hypothalamus in female P rats but not male P rats. 3α,5α-THP administration had no effects on CX3CL1 levels in both female and male VTA, hippocampus, and striatum. Detailed statistical data are presented in Table 2.

To qualitatively examine the effect of 3α,5α-THP on the intracellular distribution of CX3CL1 in the NAc of female P rats, we conducted double-immunofluorescent staining using antibodies against CX3CL1 and NeuN (a neuronal marker), CX3CL1 and TMEM119 (a microglial marker), and CX3CL1 and GFAP (an astrocyte marker). As expected, CX3CL1 was localized in NeuN-positive neuronal cells but not in TMEM119-positive microglial cells or GFAP-positive astrocytic cells, and treatment with 3α,5α-THP did not alter its intracellular localization (Figure 1b and Figure S1).

It is essential not only to measure the levels of the CX3CL1 ligand but also its specific receptor, CX3CR1, as the CX3CL1/CX3CR1 signaling pathway in the brain is associated with anti-inflammatory responses [34,42,44,93]. Furthermore, we assessed the levels of pSTAT1, a transcription factor recognized for its role in CX3CL1 production [68] and TGF-β1, considering its known influence on CX3CR1 expression and CX3CL1-mediated signaling in microglia, thereby impacting microglial–neuronal interactions and neuroinflammatory processes [65].

**Figure 2:**
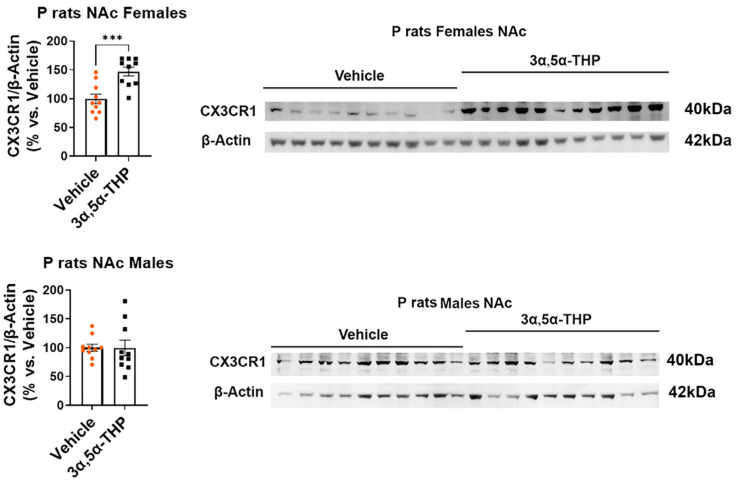
3α,5α-THP enhances CX3CR1 levels in the nucleus accumbens (NAc) of female P rats, but not in males. Male and female alcohol-preferring (P) rats (n = 10/group) were treated intraperitoneally with 3α,5α-THP (10 mg/kg) or vehicle (45% *w*/*v* 2-hydroxypropyl-β-cyclodextrin) control. After 60 min, the NAc was examined using immunoblotting assays to determine CX3CR1 expression. Specifically, in females, administering 3α,5α-THP resulted in a significant elevation of CX3CR1 levels within the NAc (*p* = 0.0006). Conversely, in male rats, treatment with 3α,5α-THP did not induce a noteworthy alteration in CX3CR1 expression within the NAc (*p* = 0.98). In the graphs, every column, along with its error bar, represents the mean ± SEM, expressed as a percentage relative to the average value of the vehicle control. Each circle represents an individual CX3CR1 value, normalized to β-Actin for vehicle-treated rats, while the black squares indicate the corresponding values for the 3α,5α-THP-treated rats. *** *p* < 0.001.

**Figure 3:**
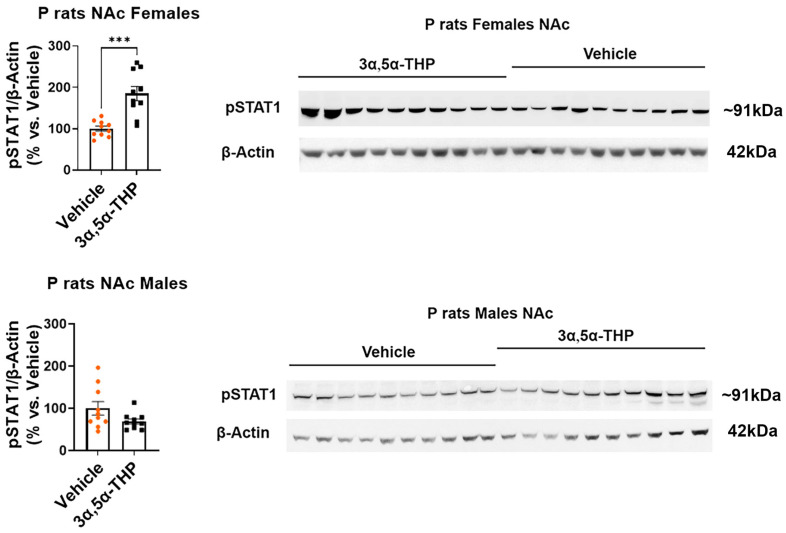
3α,5α-THP upregulates the levels of pSTAT1 in the nucleus accumbens (NAc) of female, but not male P rats. Male and female alcohol-preferring (P) rats (n = 10/group) were treated intraperitoneally with 3α,5α-THP (10 mg/kg) or vehicle (45% *w*/*v* 2-hydroxypropyl-β-cyclodextrin) control. After 60 min, the NAc was examined using immunoblotting assays to determine pSTAT1 expression. In females, the administration of 3α,5α-THP resulted in a significant increase in pSTAT1 levels within the NAc (*p* = 0.0002). However, in male rats, 3α,5α-THP treatment did not lead to a notable change in pSTAT1 expression within the NAc (*p* = 0.08). In the graphs, every column, along with its error bar, represents the mean ± SEM, expressed as a percentage relative to the average value of the vehicle control. Each circle represents an individual pSTAT1 value, normalized to β-Actin for vehicle-treated rats, while the black squares indicate the corresponding values for the 3α,5α-THP-treated rats. *** *p* < 0.001.

**Figure 4:**
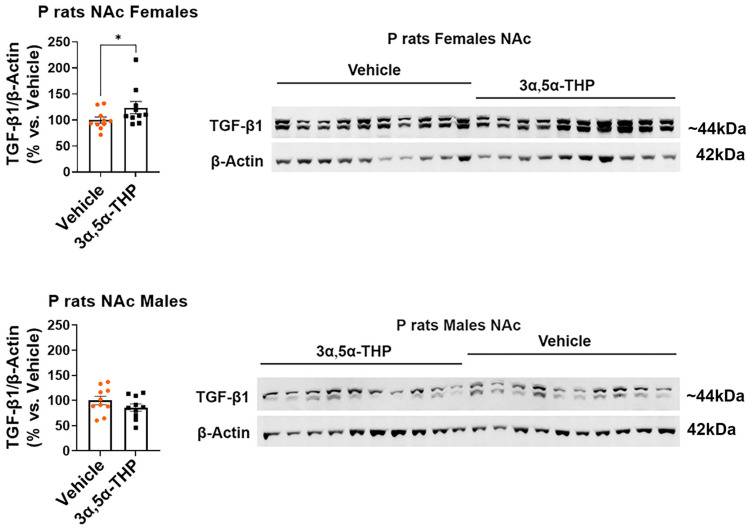
3α,5α-THP upregulates the levels of TGF-β1 in the nucleus accumbens (NAc) of female, but not male P rats. Male and female alcohol-preferring (P) rats (n = 10/group) were treated intraperitoneally with 3α,5α-THP (10 mg/kg) or vehicle (45% *w*/*v* 2-hydroxypropyl-β-cyclodextrin) control. After 60 min, the NAc was examined using immunoblotting assays to determine TGF-β1 expression. In females, the administration of 3α,5α-THP (10 mg/kg, IP) resulted in a significant increase in TGF-β1 levels within the NAc (*p* = 0.04). However, in male rats, 3α,5α-THP treatment did not lead to a notable change in TGF-β1 expression within the NAc (*p* = 0.21). In the graphs, every column, along with its error bar, represents the mean ± SEM, expressed as a percentage relative to the average value of the vehicle control. Each circle represents an individual TGF-β1 value, normalized to β-Actin for vehicle-treated rats, while the black squares indicate the corresponding values for the 3α,5α-THP-treated rats. * *p* < 0.05.

In the female P rat NAc, we observed increased levels of CX3CR1 (+47.6 ± 11.4%; *t*-test: t = 4.17, df = 18, n = 10, *p* = 0.0006) (Figure 2), pSTAT1 (+85.2 ± 17.9%; *t*-test: t = 4.74, df = 18, n = 10, *p* = 0.0002) (Figure 3), and TGF-β1 (+23.7 ± 13.3%; Mann–Whitney test: U = 26, n = 10, *p* = 0.04) (Figure 4) following 3α,5α-THP treatment. In contrast, in the male P rat NAc, there were no changes observed in the levels of CX3CR1 (*t*-test: t = 0.03, df = 18, n = 10, *p* = 0.98), pSTAT1 (*t*-test: t = 1.84, df = 18, n = 10, *p* = 0.08), or TGF-β1 (*t*-test: t = 1.29, df = 18, n = 10, *p* = 0.21) following 3α,5α-THP treatment.

### 3.2. 3α,5α-THP Downregulates the Levels of the Astrocytic Marker GFAP but Does Not Affect Microglial Markers IBA1 and TMEM119 in the Nucleus Accumbens (NAc) of Female and Male P Rats

Given the well-established role of the CX3CL1/CX3CR1 signaling axis as a pivotal functional regulator of microglia and astrocytes [29,32,34,36,37,47,53], we assessed the levels of IBA1 and TMEM119, and GFAP as markers for microglia and astrocytes, respectively. IBA1 and GFAP are known to be upregulated in reactive microglia and astrocytes, respectively [94,95], while TMEM119 is typically downregulated in reactive microglia [63,96]. Additionally, we examined the levels of CD68, CD36, TREM-2, Acrp30, and SOCS3, all of which play crucial roles in modulating brain inflammation and are predominantly expressed in microglia within the brain [67,97,98,99,100,101].

Notably, there was no significant effect observed on the expression of IBA1 and TMEM119 in the NAc of both female and male P rats (*p* > 0.05) following 3α,5α-THP treatment (Table 3). Interestingly, the levels of SOCS3 (+27.4 ± 11.7%; *t*-test: t = 2.35, df = 18, *p* = 0.03) significantly increased in the NAc of male P rats following 3α,5α-THP treatment but remained unchanged in female P rats (*p* > 0.05) (Table 3). There was no significant effect (*p* > 0.05) observed on the expression of other proteins associated with microglia, such as CD68, CD36, TREM-2, and Acrp30, in the NAc of both female and male P rats, like IBA1 and TMEM119 (Table 3).

**Figure 5:**
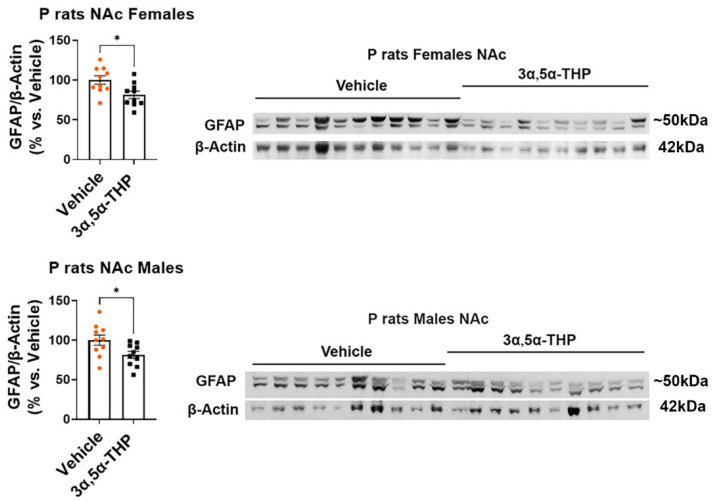
3α,5α-THP downregulates the levels of the astrocytic marker GFAP in the nucleus accumbens (NAc) of female and male P rats. Male and female alcohol-preferring (P) rats (n = 10/group) were treated intraperitoneally with 3α,5α-THP (10 mg/kg) or vehicle (45% *w*/*v* 2-hydroxypropyl-β-cyclodextrin) control. After 60 min, the NAc was examined using immunoblotting assays to determine GFAP expression. In both females (*p* = 0.02) and males (*p* = 0.03), administering 3α,5α-THP led to a significant decrease in GFAP levels within the NAc. In the graphs, every column, along with its error bar, represents the mean ± SEM, expressed as a percentage relative to the average value of the vehicle control. Each circle represents an individual GFAP value, normalized to β-Actin for vehicle-treated rats, while the black squares indicate the corresponding values for the 3α,5α-THP-treated rats. * *p* < 0.05.

However, the levels of GFAP were significantly inhibited in the NAc of both females (−18.6 ± 6.9%; *t*-test: t = 2.67, df = 18, n = 10, *p* = 0.02) and males (−18.7 ± 7.8%; *t*-test: t = 2.38, df = 18, n = 10, *p* = 0.03) following 3α,5α-THP treatment (Figure 5). These findings indicate that 3α,5α-THP primarily influences astrocyte reactivity state and does not appear to impact microglial reactivity state.

## 4. Discussion

The results of this study reveal sex-specific effects of 3α,5α-THP on CX3CL1/CX3CR1 signaling in the NAc of P rats. Specifically, in female P rats, administering 3α,5α-THP led to a significant increase in both CX3CL1 and CX3CR1 levels. However, male P rats did not show changes in these levels after treatment. Furthermore, the 3α,5α-THP-induced elevation of CX3CL1 in females, but not males, was consistent across various brain regions, including the NAc, amygdala, and hypothalamus, as well as the midbrain containing both the periaqueductal gray and the raphe nuclei. It is noteworthy that not all brain regions examined in females exhibited elevated CX3CL1 levels after 3α,5α-THP administration. Specifically, the VTA, hippocampus, and striatum in females did not show an increase in CX3CL1 expression, emphasizing the brain region-specific effect of 3α,5α-THP. Although direct statistical comparison between males and females was not conducted due to separate experimental testing, our findings elucidated significant effects of 3α,5α-THP on multiple components of the CX3CL1-CX3CR1 pathway in tissues from female, but not male, rats. This consistent replication of sex-specific effects across several independent experiments within the study underscores the strength and reliability of our observations. These findings suggest that different mechanisms may govern the modulation of the CX3CL1/CX3CR1 pathway in the male vs. female brain.

Our previous studies have demonstrated that 3α,5α-THP increases the levels of the anti-inflammatory cytokine IL-10 and BDNF in P rat brains, exhibiting a sex-specific effect by elevating their levels in males, but not females [61]. Interestingly, in human macrophages, 3α,5α-THP selectively inhibits the elevation of inflammatory cytokines IL-1β and IL-6 in macrophages obtained from female subjects, while showing no such effect in males [14]. Importantly, the CX3CL1/CX3CR1 signaling reduces the excessive production of inflammatory mediators, including these cytokines, in the brain [35,36,38,47,48,49,51,52]. Understanding the effect of 3α,5α-THP and the possible role of the CX3CL1/CX3CR1 pathway in this effect in the brain is essential due to significant roles of inflammatory mediators in initiating and perpetuating inflammatory conditions within the brain, as well as causing disruption of the blood–brain barrier and cognitive impairment [102].

The CX3CL1/CX3CR1 signaling pathway plays a pivotal role in regulating critical functions within the CNS, spanning immune responses, stress responses, cognitive processes, and pain perception. Diminished CX3CL1/CX3CR1 signaling exacerbates brain inflammation, increasing the production of inflammatory mediators and accelerating the activation of glial cells, ultimately leading to synaptic loss and heightened neurodegeneration [34,59,103,104,105]. In alcohol-preferring P rats, utilized in this study, CX3CL1 levels in the brain are innately downregulated. This downregulation is attributed to the inflammatory activation of TLR4 pathways, resulting in an imbalance between inflammatory and anti-inflammatory factors, with elevated levels of inflammatory factors such as MCP-1 and HMGB1 and decreased levels of anti-inflammatory factors such as CX3CL1 and IL-10 [59]. Likewise, activation of the TLR4 inflammatory signal leads to a decrease in the expression of its receptor, CX3CR1, within microglia [60]. It is essential to underscore that P rats, developed through selective breeding rather than genetic manipulation like knockout or mutation, represent a model closely resembling individuals not exposed to alcohol but predisposed to seek and consume it. In comparison to their alcohol-averse counterparts and wild-type rats, P rats exhibit voluntary alcohol consumption and engage in binge drinking behavior [73,74].

Therefore, the ability of 3α,5α-THP to elevate CX3CL1/CX3CR1 signaling and IL-10 production [61], coupled with its capacity to diminish inflammatory factor production in the brain [13,19], is of vital importance. These mechanisms have the potential to restore the equilibrium between inflammatory and anti-inflammatory factors in the brain, thereby offering protection against neuroinflammatory conditions commonly observed in neuropsychiatric disorders, including alcohol use disorders.

While the precise mechanisms remain unclear, it is plausible that the activation of the transcription factor STAT1 and the upregulation of TGF-β1, observed in the NAc of female P rats but not in the NAc of male P rats, could contribute to the regulation of the CX3CL1/CX3CR1 pathway. STAT1 is involved in the production of CX3CL1 by regulating its gene promoter, although it is also possible that STAT1 activation could be a consequence of CX3CL1/CX3CR1 pathway initiation [68,106]. Additionally, TGF-β1 has been shown to increase the expression of CX3CR1 in microglial cells by amplifying the transcription of its gene [65]. These processes highlight the regulatory function of STAT1 and TGF-β1 in controlling the CX3CL1/CX3CR1 pathway in the female brain.

The observed sex-specific effects of 3α,5α-THP on CX3CL1/CX3CR1 signaling could potentially be attributed to variations in sensitivity to the administered dose of 3α,5α-THP between males and females [107]. However, it is important to note that no investigations into the structural requirements for neurosteroid activity have been conducted thus far. Therefore, further studies exploring the specific structural aspects of 3α,5α-THP that mediate its effects on CX3CL1/CX3CR1 signaling are clearly warranted and may uncover additional factors contributing to the observed sex-specific responses.

In the current study, we observed an increase in SOCS3 expression induced by 3α,5α-THP in male P rat brains, a response not observed in females. Additionally, while IL-10 levels were elevated in male P rat brains following 3α,5α-THP treatment, this effect was not observed in females. The increase in IL-10 was associated with the activation of the endosomal anti-inflammatory TRIF-dependent TLR4 pathway by 3α,5α-THP [61]. SOCS3, recognized for its role in suppressing pro-inflammatory cytokine production, plays a crucial role in mitigating inflammatory responses. Its absence leads to cerebellar neutrophil infiltration and increased reactive oxygen species, ultimately resulting in brain-targeted autoimmune encephalomyelitis [108,109]. Moreover, IL-10 has been shown to induce SOCS3 expression [110,111]. Therefore, it is plausible that the elevation of SOCS3 in male P rat brains may also be attributed to the activation of the TRIF-dependent TLR4 pathway. However, further studies are warranted to validate this hypothesis. Importantly, we previously demonstrated 3α,5α-THP inhibition of this TLR4/TRIF pathway in female P rat brains, evidenced by the suppression of TRIF-related adaptor molecule activation—a specific marker for TLR4/TRIF pathway activation [61]. Thus, the upregulation of CX3CL1 in female brains is unrelated to the TLR4/TRIF pathway.

CX3CL1 emerges as a potent modulator of neuroinflammation, exerting its effects by suppressing both microglial and astrocyte activation and fostering various interactions among brain cells, which are hallmark features of the CNS immune response [29,32,34,53]. Our observations suggest that 3α,5α-THP primarily influences astrocyte reactivity. This is evidenced by the downregulation of the astrocytic marker GFAP in both female and male P rat NAc following treatment. However, we did not observe significant effects on microglial markers IBA1 and TMEM119. Additionally, 3α,5α-THP demonstrates no impact on the levels of CD68, CD36, TREM-2, and Acrp30 in the male and female NAc. These proteins are involved in regulating brain inflammation and are predominantly present in microglial cells within the brain [66,69,70,71].

The present study focused on adult P rats aged 3–4 months. However, extending this investigation to include older animals (9–12 months) and exploring the role of 3α,5α-THP is essential. Studies have shown that GFAP expression tends to increase with age, indicating heightened astrocyte reactivity in the aging brain [57,112]. Moreover, microglial activation is known to be altered in aged brains, contributing to neuroinflammation and cognitive decline [113]. Notably, endogenous 3α,5α-THP levels have been observed to decrease significantly with age in the brain [114,115]. Examining how 3α,5α-THP influences GFAP expression and microglial activation in aged brains could offer valuable insights into its therapeutic potential for age-related neurodegenerative conditions.

The inhibition of GFAP in the brain by 3α,5α-THP likely involves multiple mechanisms. One particularly promising avenue is its ability to inhibit inflammatory TLR pathways while enhancing anti-inflammatory ones. This dual action may lead to the suppression of GFAP expression by reducing levels of inflammatory factors such as MCP-1, HMGB1, TNF-α, IL-1β, and IL-6, while simultaneously increasing levels of anti-inflammatory and trophic factors like CX3CL1, IL-10, and BDNF [13,19,20,61,115]. Notably, studies have shown that GFAP levels increase in glia treated with TLR4 agonists such as lipopolysaccharide, as well as with IL-1β and TNF-α [116]. Additionally, 3α,5α-THP may influence calcium signaling, a critical regulator of GFAP expression [117,118]. Moreover, it is possible that 3α,5α-THP affects epigenetic mechanisms such as DNA methylation or histone modification, thereby influencing GFAP expression [119,120]. Investigating the precise mechanisms involved will require the further exploration of specific cellular pathways and molecular targets.

## 5. Conclusions

The findings from our study offer insights into the sex-specific and brain region-specific effects of 3α,5α-THP on CX3CL1/CX3CR1 signaling in the brain of alcohol-preferring P rats. We observe a significant increase in CX3CL1 and CX3CR1 levels in the NAc exclusively in female rats following treatment. In females, the increase in CX3CL1 extends to other brain regions such as the amygdala, midbrain (including the periaqueductal gray and the raphe nuclei), and hypothalamus. However, some brain areas, including the VTA, hippocampus, and striatum, show no effects of 3α,5α-THP on CX3CL1, indicating brain-region specificity.

The observed upregulation of pSTAT1 and TGF-β1 in females, contrasted with the absence of such effects in males, underscores potential pathways contributing to this sex-specific response. Furthermore, our study reveals a notable decrease in the astrocytic marker GFAP within the NAc of both female and male P rats following 3α,5α-THP administration, suggesting a specific influence of 3α,5α-THP on astrocyte reactivity. The absence of significant effects on microglial markers IBA1 and TMEM119, as well as CD68, CD36, TREM-2, and Acrp30, predominantly expressed in microglia in the brain, highlights the targeted impact of 3α,5α-THP on astrocytes.

The effects of 3α,5α-THP on CX3CL1/CX3CR1 signaling in the female ethanol-naïve P rat brain seem to involve both the inhibition of inflammatory TLR pathways and the restoration of balance between inflammatory and anti-inflammatory factors. The restored CX3CL1/CX3CR1 signaling, which is directly linked to the regulation of neuron–glial communication, offers potential protection against the neuroinflammatory conditions frequently observed in neuropsychiatric disorders, including alcohol use disorders.

In the brain of ethanol-naïve P rats, innate activation of inflammatory TLR pathways leads to upregulation of inflammatory factors, likely resulting in a downregulation of CX3CL1 levels in neurons and decreased CX3CR1 levels in glia, thereby diminishing CX3CL1/CX3CR1 signaling between neurons and glia. Concurrently, GFAP upregulation in astrocytes contributes to heightened neuroinflammatory conditions and disrupted neuron–glial communication.

**Figure 6:**
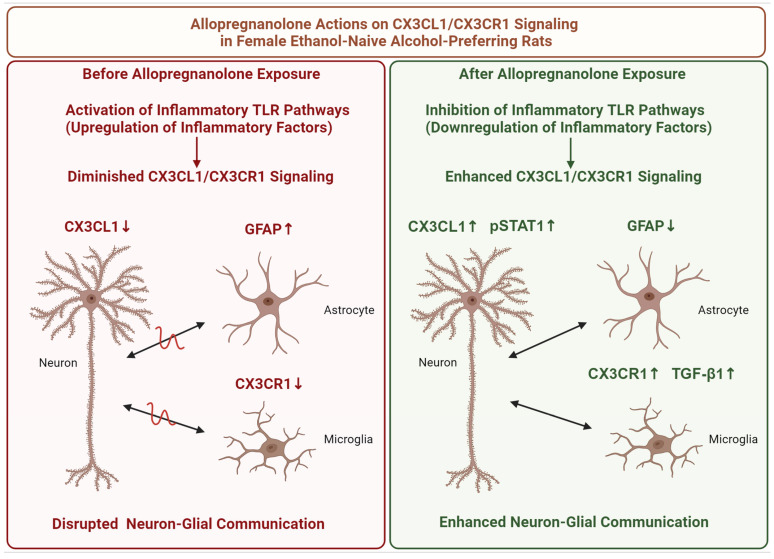
Schematic of 3α,5α-THP (allopregnanolone) actions on CX3CL1/CX3CR1 signaling in the brain of female ethanol-naïve alcohol-preferring (P) rats. Initially, activation of inflammatory TLR pathways leads to the upregulation of inflammatory factors, resulting in decreased levels (red arrows down) of CX3CL1 in neurons and CX3CR1 in glia, thereby reducing (black double-ended arrows crossed by a red zigzag) CX3CL1/CX3CR1 signaling between neurons and glia. Concurrently, GFAP upregulation (red arrow up) in astrocytes also contributes to disrupted neuron–glial communication. Upon administration of 3α,5α-THP, inflammatory TLR pathways are inhibited, resulting in the downregulation of inflammatory factors. Subsequently, CX3CL1 levels in neurons increase (green arrow up) through the activation (phosphorylation) of STAT1 (pSTAT1 upregulation is represented by a green arrow up), while CX3CR1 levels in glia are upregulated (green arrow up) via TGF-β1 (TGF-β1 upregulation is represented by a green arrow up) involvement. Additionally, GFAP levels in astrocytes decrease (green arrow down). These changes ultimately lead to enhanced neuron–glial communication (black double-ended arrows). Created with BioRender.com.

After the administration of 3α,5α-THP, inflammatory TLR pathways are inhibited, leading to the downregulation of inflammatory factors. Subsequently, there is an upregulation of CX3CL1 in neurons likely mediated by the activation of its transcription factor STAT1, while CX3CR1 in glia seems to be upregulated through the involvement of TGF-β1. Additionally, there is a concurrent downregulation of GFAP in astrocytes. We suggest that these events culminate in enhanced neuron–glial communication, potentially protecting against the neuroinflammatory conditions, as schematically illustrated in Figure 6.

In summary, our study sheds light on the intricate mechanisms by which 3α,5α-THP affects neuroinflammation and neuron–glial communication. These insights lay the basis for future investigations into the therapeutic potential of 3α,5α-THP in addressing neuroinflammatory conditions. Further research into the structural requirements and underlying mechanisms of 3α,5α-THP’s effects is warranted, offering promising avenues for the development of targeted therapies.

## 6. Patents

ALM and IB declare a U.S. provisional patent on the anti-inflammatory effects of 3α,5α-THP and related steroids for the treatment of inflammatory disease.

## Figures and Tables

**Table 1:** Clonality, host species, and dilutions of primary antibodies used in immunoblotting (IB) and immunohistochemistry (IHC). Acrp30: adipocyte complement-related protein of 30 kDa; CD36: cluster of differentiation-36; CD68: cluster of differentiation-68; GFAP: glial fibrillary acidic protein; IBA1: ionized calcium-binding adapter molecule 1; CX3CL1: CX3-C motif chemokine ligand 1 (fractalkine); CX3CR1: CX3CL1 receptor; NeuN: neuronal nuclear protein; SOCS3: suppressor of cytokine signaling 3; STAT1: signal transducer and activator of transcription 1; TGF-β1: transforming growth factor beta 1; TMEM119: transmembrane protein 119; TREM-2: triggering receptor expressed on myeloid cells 2.

Target	Catalog Number	Commercial Supplier	Clonality	Host	Dilution
CX3CL1	14-7986-81	Thermo Fisher Scientific, Waltham, MA, USA	Polyclonal	Rabbit	1:100/IHC
CX3CR1	14-6093-81	Thermo Fisher Scientific, Waltham, MA, USA	Polyclonal	Rabbit	1:1000/IB
Phospho-STAT1	9177	Cell Signaling Technology, Danvers, MA, USA	Polyclonal	Rabbit	1:1000/IB
TGF-β1	21898-1-AP	Proteintech Group, Rosemont, IL, USA	Polyclonal	Rabbit	1:2000/IB
GFAP	Z033429-2	Agilent Technologies/Dako, Santa Clara, CA, USA	Polyclonal	Rabbit	1:1000/IB
GFAP	ab4648	Abcam Inc, Cambridge, MA, USA	Monoclonal	Mouse	1:500/IHC
IBA1	MA5-27726	Thermo Fisher Scientific, Waltham, MA, USA	Monoclonal	Mouse	1:500/IB
TMEM119	400 211	Synaptic Systems GmbH, Goettingen, Germany	Monoclonal	Mouse	1:500/IB; 1:500/IHC
SOCS3	sc-73045	Santa Cruz Biotechnology, Santa Cruz, CA, USA	Monoclonal	Mouse	1:200/IB
CD68	MCA341R	Bio-Rad Laboratories, Hercules, CA, USA	Monoclonal	Mouse	1:300/IB
CD36	sc-7309	Santa Cruz Biotechnology, Santa Cruz, CA, USA	Monoclonal	Mouse	1:200/IB
Acrp30	sc-136131	Santa Cruz Biotechnology, Santa Cruz, CA, USA	Monoclonal	Mouse	1:200/IB
TREM-2	sc-373828	Santa Cruz Biotechnology, Santa Cruz, CA, USA	Monoclonal	Mouse	1:200/IB
NeuN	MAB377	Sigma-Aldrich, St. Louis, MO, USA	Monoclonal	Mouse	1:500/IHC
β-Actin	66009-1-Ig	Proteintech Group, Rosemont, IL, USA	Monoclonal	Mouse	1:3000/IB

**Table 2:** Evaluation of CX3CL1 levels by ELISA in tissue lysates obtained from the nucleus accumbens (NAc), amygdala, hypothalamus, ventral tegmental area (VTA), hippocampus, striatum, midbrain (containing both periaqueductal gray and the raphe nuclei) of female and male alcohol-preferring P rats after treatment with 3α,5α-THP or vehicle control. * *p* < 0.05, ** *p* < 0.01, *** *p* < 0.001.

Brain Area	3α,5α-THP vs. Vehicle (Mean ± SEM; pg/mg) Unpaired *t*-test: t-Value, Degrees of Freedom (df), Significance Level (*p*-Value)	
	Females	Males
NAc	1984 ± 88.2 vs. 1716 ± 52.4	2430 ± 147.0 vs. 2450 ± 85.6
	Upregulation: +15.6 ± 5.9%	No difference
	*t*-test: t = 2.61, df = 18, *p* = 0.02 *	*t*-test: t = 0.12, df = 18, *p* = 0.91
Amygdala	2635 ± 134.0 vs. 2001 ± 64.9	2579 ± 123.4 vs. 2380 ± 125.7
	Upregulation: +31.7 ± 7.4%	No difference
	*t*-test: t = 4.26, df = 18, *p* = 0.0002 ***	*t*-test: t = 1.13, df = 18, *p* = 0.27
Midbrain	1150 ± 52.8 vs. 937.9 ± 36.2	1118 ± 32.6 vs. 1124 ± 29.7
	Upregulation: +22.6 ± 6.8%	No difference
	*t*-test: t = 3.31, df = 18, *p* = 0.004 **	*t*-test: t = 0.13, df = 18, *p* = 0.90
Hypothalamus	1031 ± 21.1 vs. 838 ± 45.9	872.5 ± 54.1 vs. 929.9 ± 38.7
	Upregulation: +23.1 ± 6.0%	No difference
	*t*-test: t = 3.83, df = 18, *p* = 0.002 **	*t*-test: t = 0.80, df = 18, *p* = 0.46
VTA	2103 ± 211.3 vs. 1828 ± 153.3	1618 ± 61.5 vs. 1757 ± 157.2
	No difference	No difference
	*t*-test: t = 1.0, df = 18, *p* = 0.35	*t*-test: t = 0.82, df = 18, *p* = 0.42
Hippocampus	1567 ± 171.4 vs. 1473 ± 109.6	1721 ± 92.5 vs. 1876 ± 169.1
	No difference	No difference
	*t*-test: t = 0.43, df = 18, *p* = 0.68	*t*-test: t = 0.86, df = 18, *p* = 0.43
Striatum	1930 ± 140.2 vs. 2175 ± 208.5	1971 ± 199.5 vs. 1922 ± 232.2
	No difference	No difference
	*t*-test: t = 1.0, df = 18, *p* = 0.35	*t*-test: t = 0.16, df = 18, *p* = 0.88

**Table 3:** Evaluation using immunoblotting assays of microglial markers IBA1 and TMEM119, along with CD68, CD36, TREM-2, Acrp30, and SOCS3—known for their involvement in modulating brain inflammation—in nucleus accumbens (NAc) whole tissue lysates of female P rats did not reveal any discernible response to 3α,5α-THP. The corresponding data for NAc whole tissue lysates of male P rats is presented concurrently. * *p* < 0.05.

Marker	Percentage (%) Change Following 3α,5α-THP Administration vs. Vehicle Control Unpaired *t*-Test: t-Value, Degrees of Freedom (df), Significance Level (*p*-Value) Mann–Whitney Test: U-Value, Significance Level (*p*-Value), Sample Size (n)	
	NAc Females	NAc Males
IBA1	No difference	No difference
	t = 0.66, df = 18, *p* = 0.52	U = 26, *p* = 0.08, n = 10
TMEM119	No difference	No difference
	t = 1.00, df = 18, *p* = 0.33	t = 0.96, df = 18, *p* = 0.35
CD68	No difference	No difference
	t = 1.53, df = 18, *p* = 0.14	t = 1.75, df = 18, *p* = 0.10
CD36	No difference	No difference
	t = 0.08, df = 18, *p* = 0.94	t = 1.34, df = 18, *p* = 0.27
TREM-2	No difference	No difference
	t = 1.99, df = 18, *p* = 0.06	t = 1.24, df = 18, *p* = 0.23
Acrp30	No difference	No difference
	t = 0.16, df = 18, *p* = 0.88	t = 0.53, df = 18, *p* = 0.60
SOCS3	No difference	Upregulation: +27.4 ± 11.7%
	t = 0.38, df = 18, *p* = 0.71	t = 2.35, df = 18, *p* = 0.03 *

## Data Availability

All data is provided in the Supplementary Material.

## Supplementary Materials

The following supporting information can be downloaded at: https://www.mdpi.com/article/10.3390/life14070860/s1, Figure S1. Qualitative evaluation of 3α,5α-THP’s impact on the intracellular distribution of CX3CL1 in the NAc of female P rats.

## References

[1] Lurie D.I. (2018). An Integrative Approach to Neuroinflammation in Psychiatric disorders and Neuropathic Pain. J. Exp. Neurosci..

[2] Dunn G.A., Loftis J.M., Sullivan E.L. (2020). Neuroinflammation in psychiatric disorders: An introductory primer. Pharmacol. Biochem. Behav..

[3] Najjar S., Pearlman D.M., Alper K., Najjar A., Devinsky O. (2013). Neuroinflammation and psychiatric illness. J. Neuroinflamm..

[4] Cervellati C., Trentini A., Pecorelli A., Valacchi G. (2020). Inflammation in Neurological Disorders: The Thin Boundary between Brain and Periphery. Antioxid. Redox Signal..

[5] Boero G., Porcu P., Morrow A.L. (2020). Pleiotropic actions of allopregnanolone underlie therapeutic benefits in stress-related disease. Neurobiol. Stress.

[6] Morrow A.L., Boero G., Porcu P. (2020). A Rationale for Allopregnanolone Treatment of Alcohol Use Disorders: Basic and Clinical Studies. Alcohol. Clin. Exp. Res..

[7] Morrow A.L., Balan I., Boero G. (2022). Mechanisms Underlying Recovery From Postpartum Depression Following Brexanolone Therapy. Biol. Psychiatry.

[8] Modgil A., Parakala M.L., Ackley M.A., Doherty J.J., Moss S.J., Davies P.A. (2017). Endogenous and synthetic neuroactive steroids evoke sustained increases in the efficacy of GABAergic inhibition via a protein kinase C-dependent mechanism. Neuropharmacology.

[9] Stell B.M., Brickley S.G., Tang C.Y., Farrant M., Mody I. (2003). Neuroactive steroids reduce neuronal excitability by selectively enhancing tonic inhibition mediated by delta subunit-containing GABAA receptors. Proc. Natl. Acad. Sci. USA.

[10] Pinna G. (2020). Allopregnanolone (1938–2019): A trajectory of 80 years of outstanding scientific achievements. Neurobiol. Stress.

[11] Pinna G. (2020). Allopregnanolone, the Neuromodulator Turned Therapeutic Agent: Thank You, Next?. Front. Endocrinol..

[12] Antonoudiou P., Colmers P.L.W., Walton N.L., Weiss G.L., Smith A.C., Nguyen D.P., Lewis M., Quirk M.C., Barros L., Melon L.C. (2022). Allopregnanolone Mediates Affective Switching Through Modulation of Oscillatory States in the Basolateral Amygdala. Biol. Psychiatry.

[13] Balan I., Beattie M.C., O’Buckley T.K., Aurelian L., Morrow A.L. (2019). Endogenous Neurosteroid (3⍺,5⍺)3-Hydroxypregnan-20-one Inhibits Toll-like-4 Receptor Activation and Pro-inflammatory Signaling in Macrophages and Brain. Sci. Rep..

[14] Balan I., Aurelian L., Williams K.S., Campbell B., Meeker R.B., Morrow A.L. (2022). Inhibition of human macrophage activation via pregnane neurosteroid interactions with toll-like receptors: Sex differences and structural requirements. Front. Immunol..

[15] Langmade S.J., Gale S.E., Frolov A., Mohri I., Suzuki K., Mellon S.H., Walkley S.U., Covey D.F., Schaffer J.E., Ory D.S. (2006). Pregnane X receptor (PXR) activation: A mechanism for neuroprotection in a mouse model of Niemann-Pick C disease. Proc. Natl. Acad. Sci. USA.

[16] Meltzer-Brody S., Colquhoun H., Riesenberg R., Epperson C.N., Deligiannidis K.M., Rubinow D.R., Li H., Sankoh A.J., Clemson C., Schacterle A. (2018). Brexanolone injection in post-partum depression: Two multicentre, double-blind, randomised, placebo-controlled, phase 3 trials. Lancet.

[17] Balan I., Patterson R., Boero G., Krohn H., O’Buckley T.K., Meltzer-Brody S., Morrow A.L. (2023). Brexanolone therapeutics in post-partum depression involves inhibition of systemic inflammatory pathways. EBioMedicine.

[18] Patterson R., Balan I., Morrow A.L., Meltzer-Brody S. (2024). Novel neurosteroid therapeutics for post-partum depression: Perspectives on clinical trials, program development, active research, and future directions. Neuropsychopharmacology.

[19] Balan I., Aurelian L., Schleicher R., Boero G., O’Buckley T., Morrow A.L. (2021). Neurosteroid allopregnanolone (3alpha,5alpha-THP) inhibits inflammatory signals induced by activated MyD88-dependent toll-like receptors. Transl. Psychiatry.

[20] Murugan S., Jakka P., Namani S., Mujumdar V., Radhakrishnan G. (2019). The neurosteroid pregnenolone promotes degradation of key proteins in the innate immune signaling to suppress inflammation. J. Biol. Chem..

[21] Su L., Sun Y., Ma F., Lü P., Huang H., Zhou J. (2009). Progesterone inhibits Toll-like receptor 4-mediated innate immune response in macrophages by suppressing NF-kappaB activation and enhancing SOCS1 expression. Immunol. Lett..

[22] Jones L.A., Anthony J.P., Henriquez F.L., Lyons R.E., Nickdel M.B., Carter K.C., Alexander J., Roberts C.W. (2008). Toll-like receptor-4-mediated macrophage activation is differentially regulated by progesterone via the glucocorticoid and progesterone receptors. Immunology.

[23] Zhu Y., Wu M., Wu C.Y., Xia G.Q. (2013). Role of progesterone in TLR4-MyD88-dependent signaling pathway in pre-eclampsia. J. Huazhong Univ. Sci. Technol. Med. Sci..

[24] Zandieh Z., Amjadi F., Ashrafi M., Aflatoonian A., Fazeli A., Aflatoonian R. (2016). The Effect of Estradiol and Progesterone on Toll Like Receptor Gene Expression in A Human Fallopian Tube Epithelial Cell Line. Cell J..

[25] Chen G., Shi J., Jin W., Wang L., Xie W., Sun J., Hang C. (2008). Progesterone administration modulates TLRs/NF-kappaB signaling pathway in rat brain after cortical contusion. Ann. Clin. Lab. Sci..

[26] Naert G., Maurice T., Tapia-Arancibia L., Givalois L. (2007). Neuroactive steroids modulate HPA axis activity and cerebral brain-derived neurotrophic factor (BDNF) protein levels in adult male rats. Psychoneuroendocrinology.

[27] Nin M.S., Martinez L.A., Pibiri F., Nelson M., Pinna G. (2011). Neurosteroids reduce social isolation-induced behavioral deficits: A proposed link with neurosteroid-mediated upregulation of BDNF expression. Front. Endocrinol..

[28] Almeida F.B., Nin M.S., Barros H.M.T. (2020). The role of allopregnanolone in depressive-like behaviors: Focus on neurotrophic proteins. Neurobiol. Stress.

[29] Harrison J.K., Jiang Y., Chen S., Xia Y., Maciejewski D., McNamara R.K., Streit W.J., Salafranca M.N., Adhikari S., Thompson D.A. (1998). Role for neuronally derived fractalkine in mediating interactions between neurons and CX3CR1-expressing microglia. Proc. Natl. Acad. Sci. USA.

[30] Tarozzo G., Bortolazzi S., Crochemore C., Chen S.-C., Lira A.S., Abrams J.S., Beltramo M. (2003). Fractalkine protein localization and gene expression in mouse brain. J. Neurosci. Res..

[31] Mizutani M., Pino P.A., Saederup N., Charo I.F., Ransohoff R.M., Cardona A.E. (2012). The Fractalkine Receptor but Not CCR2 Is Present on Microglia from Embryonic Development throughout Adulthood. J. Immunol..

[32] Maciejewski-Lenoir D., Chen S., Feng L., Maki R., Bacon K.B. (1999). Characterization of fractalkine in rat brain cells: Migratory and activation signals for CX3CR-1-expressing microglia. J. Immunol..

[33] Lauro C., Catalano M., Di Paolo E., Chece G., de Costanzo I., Trettel F., Limatola C. (2015). Fractalkine/CX3CL1 engages different neuroprotective responses upon selective glutamate receptor overactivation. Front. Cell. Neurosci..

[34] Kawamura N., Katsuura G., Yamada-Goto N., Nakama R., Kambe Y., Miyata A., Furuyashiki T., Narumiya S., Ogawa Y., Inui A. (2022). Brain fractalkine-CX3CR1 signalling is anti-obesity system as anorexigenic and anti-inflammatory actions in diet-induced obese mice. Sci. Rep..

[35] Bérangère Ré D., Przedborski S. (2006). Fractalkine: Moving from chemotaxis to neuroprotection. Nat. Neurosci..

[36] Pabon M.M., Bachstetter A.D., Hudson C.E., Gemma C., Bickford P.C. (2011). CX3CL1 reduces neurotoxicity and microglial activation in a rat model of Parkinson’s disease. J. Neuroinflamm..

[37] Zhao J., Li Q., Ouyang X., Wang F., Li Q., Xu Z., Ji D., Wu Q., Zhang J., Lu C. (2023). The effect of CX3CL1/ CX3CR1 signal axis on microglia in central nervous system diseases. J. Neurorestoratol..

[38] Morganti J.M., Nash K.R., Grimmig B.A., Ranjit S., Small B., Bickford P.C., Gemma C. (2012). The Soluble Isoform of CX3CL1 Is Necessary for Neuroprotection in a Mouse Model of Parkinson’s Disease. J. Neurosci..

[39] Xiao F., Xu J.M., Jiang X.H. (2015). CX3 chemokine receptor 1 deficiency leads to reduced dendritic complexity and delayed maturation of newborn neurons in the adult mouse hippocampus. Neural. Regen. Res..

[40] Noda M., Doi Y., Liang J., Kawanokuchi J., Sonobe Y., Takeuchi H., Mizuno T., Suzumura A. (2011). Fractalkine attenuates excito-neurotoxicity via microglial clearance of damaged neurons and antioxidant enzyme heme oxygenase-1 expression. J. Biol. Chem..

[41] Meucci O., Fatatis A., Simen A.A., Miller R.J. (2000). Expression of CX3CR1 chemokine receptors on neurons and their role in neuronal survival. Proc. Natl. Acad. Sci. USA.

[42] Castro-Sánchez S., García-Yagüe Á.J., López-Royo T., Casarejos M., Lanciego J.L., Lastres-Becker I. (2018). Cx3cr1-deficiency exacerbates alpha-synuclein-A53T induced neuroinflammation and neurodegeneration in a mouse model of Parkinson’s disease. Glia.

[43] Dorfman M.D., Krull J.E., Douglass J.D., Fasnacht R., Lara-Lince F., Meek T.H., Shi X., Damian V., Nguyen H.T., Matsen M.E. (2017). Sex differences in microglial CX3CR1 signalling determine obesity susceptibility in mice. Nat. Commun..

[44] Cardona S.M., Kim S.V., Church K.A., Torres V.O., Cleary I.A., Mendiola A.S., Saville S.P., Watowich S.S., Parker-Thornburg J., Soto-Ospina A. (2018). Role of the Fractalkine Receptor in CNS Autoimmune Inflammation: New Approach Utilizing a Mouse Model Expressing the Human CX3CR1^I249/M280^ Variant. Front. Cell. Neurosci..

[45] Nash K.R., Moran P., Finneran D.J., Hudson C., Robinson J., Morgan D., Bickford P.C. (2015). Fractalkine over expression suppresses α-synuclein-mediated neurodegeneration. Mol. Ther..

[46] Paolicelli R.C., Bisht K., Tremblay M. (2014). Fractalkine regulation of microglial physiology and consequences on the brain and behavior. Front. Cell. Neurosci..

[47] Zujovic V., Benavides J., Vigé X., Carter C., Taupin V. (2000). Fractalkine modulates TNF-alpha secretion and neurotoxicity induced by microglial activation. Glia.

[48] Mizuno T., Kawanokuchi J., Numata K., Suzumura A. (2003). Production and neuroprotective functions of fractalkine in the central nervous system. Brain Res..

[49] Cardona A.E., Pioro E.P., Sasse M.E., Kostenko V., Cardona S.M., Dijkstra I.M., Huang D., Kidd G., Dombrowski S., Dutta R. (2006). Control of microglial neurotoxicity by the fractalkine receptor. Nat. Neurosci..

[50] Sokolowski J.D., Chabanon-Hicks C.N., Han C.Z., Heffron D.S., Mandell J.W. (2014). Fractalkine is a “find-me” signal released by neurons undergoing ethanol-induced apoptosis. Front. Cell. Neurosci..

[51] Zujovic V., Schussler N., Jourdain D., Duverger D., Taupin V. (2001). In vivo neutralization of endogenous brain fractalkine increases hippocampal TNFalpha and 8-isoprostane production induced by intracerebroventricular injection of LPS. J. Neuroimmunol..

[52] Bachstetter A.D., Morganti J.M., Jernberg J., Schlunk A., Mitchell S.H., Brewster K.W., Hudson C.E., Cole M.J., Harrison J.K., Bickford P.C. (2011). Fractalkine and CX 3 CR1 regulate hippocampal neurogenesis in adult and aged rats. Neurobiol. Aging.

[53] Milinkeviciute G., Chokr S.M., Castro E.M., Cramer K.S. (2021). CX3CR1 mutation alters synaptic and astrocytic protein expression, topographic gradients, and response latencies in the auditory brainstem. J. Comp. Neurol..

[54] Escartin C., Galea E., Lakatos A., O’Callaghan J.P., Petzold G.C., Serrano-Pozo A., Steinhäuser C., Volterra A., Carmignoto G., Agarwal A. (2021). Reactive astrocyte nomenclature, definitions, and future directions. Nat. Neurosci..

[55] Escartin C., Guillemaud O., Carrillo-de Sauvage M.A. (2019). Questions and (some) answers on reactive astrocytes. Glia.

[56] Ingelsson M., Fukumoto H., Newell K.L., Growdon J.H., Hedley–Whyte E.T., Frosch M.P., Albert M.S., Hyman B.T., Irizarry M.C. (2004). Early Aβ accumulation and progressive synaptic loss, gliosis, and tangle formation in AD brain. Neurology.

[57] Wruck W., Adjaye J. (2020). Meta-analysis of human prefrontal cortex reveals activation of GFAP and decline of synaptic transmission in the aging brain. Acta Neuropathol. Commun..

[58] Abdelhak A., Foschi M., Abu-Rumeileh S., Yue J.K., D’Anna L., Huss A., Oeckl P., Ludolph A.C., Kuhle J., Petzold A. (2022). Blood GFAP as an emerging biomarker in brain and spinal cord disorders. Nat. Rev. Neurol..

[59] Aurelian L., Balan I. (2019). GABA_A_R α2-activated neuroimmune signal controls binge drinking and impulsivity through regulation of the CCL2/CX3CL1 balance. Psychopharmacology.

[60] Wynne A.M., Henry C.J., Huang Y., Cleland A., Godbout J.P. (2010). Protracted downregulation of CX3CR1 on microglia of aged mice after lipopolysaccharide challenge. Brain Behav. Immun..

[61] Balan I., Grusca A., O’Buckley T.K., Morrow A.L. (2023). Neurosteroid [3α,5α]-3-hydroxy-pregnan-20-one enhances IL-10 production via endosomal TRIF-dependent TLR4 signaling pathway. Front. Endocrinol..

[62] Roche S.L., Wyse-Jackson A.C., Ruiz-Lopez A.M., Byrne A.M., Cotter T.G. (2017). Fractalkine-CX3CR1 signaling is critical for progesterone-mediated neuroprotection in the retina. Sci. Rep..

[63] Ruan C., Elyaman W. (2022). A New Understanding of TMEM119 as a Marker of Microglia. Front. Cell. Neurosci..

[64] Du X., Penalva R., Little K., Kissenpfennig A., Chen M., Xu H. (2021). Deletion of *Socs3* in LysM^+^ cells and *Cx3cr1* resulted in age-dependent development of retinal microgliopathy. Mol. Neurodegener..

[65] Chen S., Luo D., Streit W.J., Harrison J.K. (2002). TGF-beta1 upregulates CX3CR1 expression and inhibits fractalkine-stimulated signaling in rat microglia. J. Neuroimmunol..

[66] Hopperton K.E., Mohammad D., Trépanier M.O., Giuliano V., Bazinet R.P. (2018). Markers of microglia in post-mortem brain samples from patients with Alzheimer’s disease: A systematic review. Mol. Psychiatry.

[67] Chakrabarti S., Jana M., Roy A., Pahan K. (2018). Upregulation of Suppressor of Cytokine Signaling 3 in Microglia by Cinnamic Acid. Curr. Alzheimer Res..

[68] Gan A.M., Butoi E., Manea A., Pirvulescu M.M., Stan D., Simion V., Calin M., Simionescu M., Manduteanu I. (2014). Functional analysis of the fractalkine gene promoter in human aortic smooth muscle cells exposed to proinflammatory conditions. FEBS J..

[69] Coraci I.S., Husemann J., Berman J.W., Hulette C., Dufour J.H., Campanella G.K., Luster A.D., Silverstein S.C., El-Khoury J.B. (2002). CD36, a class B scavenger receptor, is expressed on microglia in Alzheimer’s disease brains and can mediate production of reactive oxygen species in response to beta-amyloid fibrils. Am. J. Pathol..

[70] Nicolas S., Cazareth J., Zarif H., Guyon A., Heurteaux C., Chabry J., Petit-Paitel A. (2017). Globular Adiponectin Limits Microglia Pro-Inflammatory Phenotype through an AdipoR1/NF-κB Signaling Pathway. Front. Cell. Neurosci..

[71] Qu W., Li L. (2023). Microglial TREM2 at the Intersection of Brain Aging and Alzheimer’s Disease. Neuroscientist.

[72] Pirvulescu M., Manduteanu I., Gan A.M., Stan D., Simion V., Butoi E., Calin M., Simionescu M. (2012). A novel pro-inflammatory mechanism of action of resistin in human endothelial cells: Up-regulation of SOCS3 expression through STAT3 activation. Biochem. Biophys. Res. Commun..

[73] Beckwith S.W., Czachowski C.L. (2016). Alcohol-Preferring P Rats Exhibit Elevated Motor Impulsivity Concomitant with Operant Responding and Self-Administration of Alcohol. Alcohol. Clin. Exp. Res..

[74] Bell R.L., Rodd Z.A., Lumeng L., Murphy J.M., McBride W.J. (2006). The alcohol-preferring P rat and animal models of excessive alcohol drinking. Addict. Biol..

[75] Liu J., Yang A.R., Kelly T., Puche A., Esoga C., June H.L., Elnabawi A., Merchenthaler I., Sieghart W., June H.L. (2011). Binge alcohol drinking is associated with GABAA alpha2-regulated Toll-like receptor 4 (TLR4) expression in the central amygdala. Proc. Natl. Acad. Sci. USA.

[76] Balan I., Warnock K.T., Puche A., Gondre-Lewis M.C., June H., Aurelian L. (2018). The GABA_A_ Receptor α2 Subunit Activates a Neuronal TLR4 Signal in the Ventral Tegmental Area that Regulates Alcohol and Nicotine Abuse. Brain Sci..

[77] Bach P., Luderer M., Müller U.J., Jakobs M., Baldermann J.C., Voges J., Kiening K., Lux A., Visser-Vandewalle V., Klosterkötter J. (2023). Deep brain stimulation of the nucleus accumbens in treatment-resistant alcohol use disorder: A double-blind randomized controlled multi-center trial. Transl. Psychiatry.

[78] Bracht T., Soravia L., Moggi F., Stein M., Grieder M., Federspiel A., Tschümperlin R., Batschelet H.M., Wiest R., Denier N. (2021). The role of the orbitofrontal cortex and the nucleus accumbens for craving in alcohol use disorder. Transl. Psychiatry.

[79] Roberto M., Kirson D., Khom S. (2021). The Role of the Central Amygdala in Alcohol Dependence. Cold Spring Harb. Perspect. Med..

[80] June H.L., Liu J., Warnock K.T., Bell K.A., Balan I., Bollino D., Puche A., Aurelian L. (2015). CRF-amplified neuronal TLR4/MCP-1 signaling regulates alcohol self-administration. Neuropsychopharmacology.

[81] Dunne N., Ivers J.-H. (2023). HPA axis function in alcohol use disorder: A systematic review and meta-analysis. Addict. Neurosci..

[82] Avegno E.M., Gilpin N.W. (2022). Reciprocal midbrain-extended amygdala circuit activity in preclinical models of alcohol use and misuse. Neuropharmacology.

[83] Mira R.G., Lira M., Tapia-Rojas C., Rebolledo D.L., Quintanilla R.A., Cerpa W. (2019). Effect of Alcohol on Hippocampal-Dependent Plasticity and Behavior: Role of Glutamatergic Synaptic Transmission. Front. Behav. Neurosci..

[84] Sitzia G., Lovinger D.M. (2023). Circuit dysfunctions of associative and sensorimotor basal ganglia loops in alcohol use disorder: Insights from animal models. Addict. Neurosci..

[85] Vázquez-León P., Miranda-Páez A., Chávez-Reyes J., Allende G., Barragán-Iglesias P., Marichal-Cancino B.A. (2021). The Periaqueductal Gray and Its Extended Participation in Drug Addiction Phenomena. Neurosci. Bull..

[86] Belmer A., Depoortere R., Beecher K., Newman-Tancredi A., Bartlett S.E. (2022). Neural serotonergic circuits for controlling long-term voluntary alcohol consumption in mice. Mol. Psychiatry.

[87] Ogle T.F., Kitay J.I. (1977). Ovarian and adrenal steroids during pregnancy and the oestrous cycle in the rat. J. Endocrinol..

[88] Sze Y., Brunton P.J. (2020). Sex, stress and steroids. Eur. J. Neurosci..

[89] Heffner T.G., Hartman J.A., Seiden L.S. (1980). A rapid method for the regional dissection of the rat brain. Pharmacol. Biochem. Behav..

[90] Mendelson W.B., Martin J.V., Perlis M., Wagner R., Majewska M.D., Paul S.M. (1987). Sleep induction by an adrenal steroid in the rat. Psychopharmacology.

[91] Devaud L.L., Purdy R.H., Morrow A.L. (1995). The neurosteroid, 3α-hydroxy-5α-pregnan-20-one, protects against bicuculline-induced seizures during ethanol withdrawal in rats. Alcohol. Clin. Exp. Res..

[92] Crawley J.N., Glowa J.R., Majewska M.D., Paul S.M. (1986). Anxiolytic activity of an endogenous adrenal steroid. Brain Res..

[93] Inoue K., Morimoto H., Ohgidani M., Ueki T. (2021). Modulation of inflammatory responses by fractalkine signaling in microglia. PLoS ONE.

[94] Jurga A.M., Paleczna M., Kuter K.Z. (2020). Overview of General and Discriminating Markers of Differential Microglia Phenotypes. Front. Cell. Neurosci..

[95] Gomes F.C., Paulin D., Moura Neto V. (1999). Glial fibrillary acidic protein (GFAP): Modulation by growth factors and its implication in astrocyte differentiation. Braz. J. Med. Biol. Res..

[96] Mercurio D., Fumagalli S., Schafer M.K., Pedragosa J., Ngassam L.D.C., Wilhelmi V., Winterberg S., Planas A.M., Weihe E., De Simoni M.G. (2022). Protein Expression of the Microglial Marker Tmem119 Decreases in Association with Morphological Changes and Location in a Mouse Model of Traumatic Brain Injury. Front. Cell. Neurosci..

[97] Yeo H.G., Hong J.J., Lee Y., Yi K.S., Jeon C.Y., Park J., Won J., Seo J., Ahn Y.J., Kim K. (2019). Increased CD68/TGFβ Co-expressing Microglia/ Macrophages after Transient Middle Cerebral Artery Occlusion in Rhesus Monkeys. Exp. Neurobiol..

[98] Yang J., Fu Z., Zhang X., Xiong M., Meng L., Zhang Z. (2020). TREM2 ectodomain and its soluble form in Alzheimer’s disease. J. Neuroinflamm..

[99] Takahashi K., Rochford C.D., Neumann H. (2005). Clearance of apoptotic neurons without inflammation by microglial triggering receptor expressed on myeloid cells-2. J. Exp. Med..

[100] Dobri A.-M., Dudău M., Enciu A.-M., Hinescu M.E. (2021). CD36 in Alzheimer’s Disease: An Overview of Molecular Mechanisms and Therapeutic Targeting. Neuroscience.

[101] Song J., Choi S.M., Kim B.C. (2017). Adiponectin Regulates the Polarization and Function of Microglia via PPAR-γ Signaling Under Amyloid β Toxicity. Front. Cell. Neurosci..

[102] Yang J., Ran M., Li H., Lin Y., Ma K., Yang Y., Fu X., Yang S. (2022). New insight into neurological degeneration: Inflammatory cytokines and blood-brain barrier. Front. Mol. Neurosci..

[103] Sheridan G.K., Wdowicz A., Pickering M., Watters O., Halley P., O’Sullivan N.C., Mooney C., O’Connell D.J., O’Connor J.J., Murphy K.J. (2014). CX3CL1 is up-regulated in the rat hippocampus during memory-associated synaptic plasticity. Front. Cell. Neurosci..

[104] Kawamura N., Katsuura G., Yamada-Goto N., Novianti E., Inui A., Asakawa A. (2020). Reduced brain fractalkine-CX3CR1 signaling is involved in the impaired cognition of streptozotocin-treated mice. IBRO Rep..

[105] Réaux-Le Goazigo A., Van Steenwinckel J., Rostène W., Mélik Parsadaniantz S. (2013). Current status of chemokines in the adult CNS. Prog. Neurobiol..

[106] Imaizumi T., Yoshida H., Satoh K. (2004). Regulation of CX3CL1/Fractalkine Expression in Endothelial Cells. J. Atheroscler. Thromb..

[107] Morrow A.L., Boero G., Balan I. (2024). Emerging Evidence for Endogenous Neurosteroid Modulation of Pro-Inflammatory and Anti-Inflammatory Pathways that Impact Neuropsychiatric Disease. Neurosci. Biobehav. Rev..

[108] Yan Z., Yang W., Parkitny L., Gibson S.A., Lee K.S., Collins F., Deshane J.S., Cheng W., Weinmann A.S., Wei H. (2019). Deficiency of Socs3 leads to brain-targeted EAE via enhanced neutrophil activation and ROS production. JCI Insight.

[109] Turbitt W.J., Yan Z., Yang W., Buckley J., Zhou L., Qin H., Benveniste E. (2021). Neutrophil-specific Socs3 deficiency induces brain-targeted experimental autoimmune encephalomyelitis with increased cerebellar neutrophils. J. Immunol..

[110] Cassatella M.A., Gasperini S., Bovolenta C., Calzetti F., Vollebregt M., Scapini P., Marchi M., Suzuki R., Suzuki A., Yoshimura A. (1999). Interleukin-10 (IL-10) Selectively Enhances CIS3/SOCS3 mRNA Expression in Human Neutrophils: Evidence for an IL-10–Induced Pathway That Is Independent of STAT Protein Activation. Blood.

[111] Cevey Á.C., Penas F.N., Alba Soto C.D., Mirkin G.A., Goren N.B. (2019). IL-10/STAT3/SOCS3 Axis Is Involved in the Anti-inflammatory Effect of Benznidazole. Front. Immunol..

[112] Nichols N.R., Day J.R., Laping N.J., Johnson S.A., Finch C.E. (1993). GFAP mRNA increases with age in rat and human brain. Neurobiol. Aging.

[113] Antignano I., Liu Y., Offermann N., Capasso M. (2023). Aging microglia. Cell Mol. Life Sci..

[114] Bernardi F., Salvestroni C., Casarosa E., Nappi R.E., Lanzone A., Luisi S., Purdy R.H., Petraglia F., Genazzani A.R. (1998). Aging is associated with changes in allopregnanolone concentrations in brain, endocrine glands and serum in male rats. Eur. J. Endocrinol..

[115] Balan I., Boero G., Chéry S.L., McFarland M.H., Lopez A.G., Morrow A.L. (2024). Neuroactive Steroids, Toll-like Receptors, and Neuroimmune Regulation: Insights into Their Impact on Neuropsychiatric Disorders. Life.

[116] von Boyen G.B., Steinkamp M., Reinshagen M., Schäfer K.H., Adler G., Kirsch J. (2004). Proinflammatory cytokines increase glial fibrillary acidic protein expression in enteric glia. Gut.

[117] Wang T., Chen S., Mao Z., Shang Y., Brinton R.D. (2023). Allopregnanolone pleiotropic action in neurons and astrocytes: Calcium signaling as a unifying mechanism. Front. Endocrinol..

[118] Lee Y.B., Du S., Rhim H., Lee E.B., Markelonis G.J., Oh T.H. (2000). Rapid increase in immunoreactivity to GFAP in astrocytes in vitro induced by acidic pH is mediated by calcium influx and calpain I. Brain Res..

[119] Colciago A., Magnaghi V. (2016). Neurosteroids Involvement in the Epigenetic Control of Memory Formation and Storage. Neural Plast..

[120] Kumar P., Godbole N.M., Chaturvedi C.P., Singh R.S., George N., Upadhyay A., Anjum B., Godbole M.M., Sinha R.A. (2018). Mechanisms involved in epigenetic down-regulation of Gfap under maternal hypothyroidism. Biochem. Biophys. Res. Commun..

