# Supplementary material for: Neurosteroid [3α,5α]-3-Hydroxy-pregnan-20-one Enhances the CX3CL1-CX3CR1 Pathway in the Brain of Alcohol-Preferring Rats with Sex-Specificity
Source: Life (Basel). 2024 Jul 9;14(7):860. doi: 10.3390/life14070860 (PMC11277648; doi:10.3390/life14070860)
Supplement: Supplementary file 1 [file life-14-00860-s001.zip › life-3005160-supplementary.pdf]

Supplementary Materials

Neurosteroid [3α,5α]-3-Hydroxy-pregnan-20-one Enhances the CX3CL1-CX3CR1 Pathway in the Brain of Alcohol-Preferring Rats with Sex-Specificity

Irina Balan, Adelina Grusca, Samantha Lucenell Chéry, Baylee R. Materia, Todd K. O'Buckley and A. Leslie Morrow

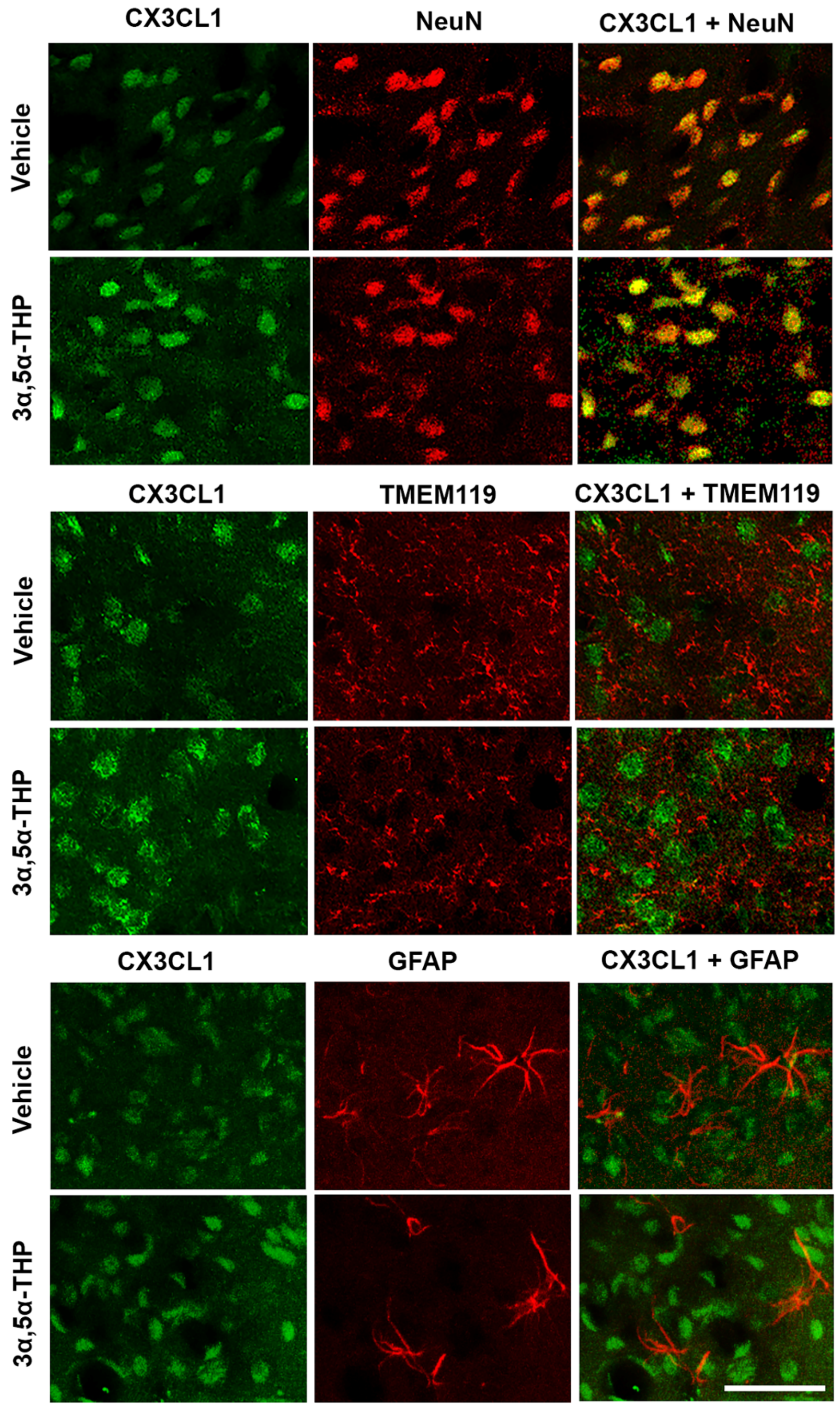

Figure S1. Qualitative evaluation of 3α,5α-THP's impact on the intracellular distribution of CX3CL1 in the NAc of female P rats. Double-immunofluorescent staining was conducted using antibodies targeting CX3CL1 alongside NeuN (a neuronal marker), TMEM119 (a microglial marker) or GFAP (an astrocyte marker). In vehicle control, CX3CL1 was observed to localize within NeuN-positive neuronal cells, but not co-localizing with TMEM119-positive microglial cells or GFAP-positive astrocytic cells. Treatment with 3α,5α-THP did not induce any evident alteration in the CX3CL1 intracellular localization. Scale bar is 50 μm.
